# Agonistic activity of tamoxifen, a selective estrogen-receptor modulator (SERM), on arthritic ovariectomized mice

**DOI:** 10.1590/1414-431X20176799

**Published:** 2017-11-13

**Authors:** L.A.S. Silva, F.B. Felix, J.M.D. Araujo, E.V. Souza, E.A. Camargo, R. Grespan

**Affiliations:** Programa de Pós-Graduação em Ciências Fisiológicas, Departamento de Fisiologia, Universidade Federal de Sergipe, São Cristóvão, SE, Brasil

**Keywords:** Arthritis, Tamoxifen, Neutrophils, Estrogen, Ovariectomy

## Abstract

Arthritis is positively associated with the decline of sex hormones, especially estrogen. Tamoxifen (TMX) is a selective estrogen receptor modulator, possessing agonist or antagonistic activity in different tissues. Thus, the objective of this study was to investigate the effect of TMX on the zymosan-induced arthritis model. Female Swiss normal and ovariectomized (OVX) mice were divided into groups and treated for five days with TMX (0.3, 0.9 or 2.7 mg/kg) or 17-β-estradiol (E2, 50 µg/kg). On the fifth day, arthritis was induced and 4 h later, leukocyte migration into joint cavities was evaluated. The neutrophil migration in OVX animals, but not in normal mice, treated with TMX (all tested doses) was significantly decreased compared with mice that received the vehicle (P≤0.05). Similarly, this effect was also demonstrated in the E2-treated group. Therefore, the present study demonstrates that TMX presented agonist effects in inhibiting neutrophil migration and preventing arthritis progression in OVX mice.

## Introduction

Arthritis is characterized by persistent synovitis and systemic inflammation, being more prevalent among women than men, especially during the postmenopausal phase. It is associated positively with the decline of sex hormones, mainly estrogen (1).

During menopause, the decrease in estrogen levels potentially increases the vulnerability to diseases such as cancer, arthritis and osteoporosis in hormone-responsive tissues (2). Furthermore, it is known that the estrogen receptor alpha is expressed in synovial tissues, and that it is regulated upwards in the presence of inflammation (1).

The acute inflammation phase of arthritis has been well mimicked by the intra-articular injection of zymosan, which is accompanied by an increased profile of proinflammatory cytokines, such as interleukin-1 (IL-1), tumor necrosis factor alpha (TNF-α) and IL-6, prominent infiltration of neutrophils and joint swelling that are used to investigate the effects of a variety of drugs (3).

Several studies have focused on the activity of the selective estrogen receptor modulators (SERMs) in different tissues (4,5). SERMs have been used for decades in the study of estrogen receptor antagonism or agonism (6). In the treatment of breast cancer by tamoxifen (TMX), SERMs act as antagonists, but several side effects are reported by most patients, related to menopausal symptoms, including joint pain (7).

In contrast, TMX presents estrogen agonist activity in several tissues, such as uterus, bone and liver (8). A previous study showed that TMX prevents the increase in bone resorption at the medullary surface in ovariectomized rats (9). In addition, it suppresses the protein hypoxia-inducible factor 1 alpha (HIF1-α) in osteoclasts responsible for developing osteoporosis, similarly to estrogen (10).

Therefore, it is important to know the effect of tamoxifen on experimental arthritis in order to develop new treatment strategies. Thus, the objective of this study was to investigate the effect of tamoxifen on zymosan-induced arthritis model.

## Material and Methods

### Animals

Swiss female mice (25–30 g) were provided by the Animal House of the Federal University of Sergipe. The animals were housed at a controlled temperature (22±1°C) in a 12-h light-dark cycle with free access to food and water. All protocols were approved by the Ethics Committee for Animal Experimentation of the Universidade Federal de Sergipe under protocol No. 10/2015 and were conducted in compliance with the Guide for Care and Use of Laboratory Animals (National Institutes of Health). For the experiments, animals were randomly distributed in groups and the experimenter was unaware of the group's identification.

### Experimental protocol

The mice were randomly divided into 6 experimental groups with 10 animals per group: group 1, negative control group received intra-articularly (*ia*) saline solution (0.9%); group 2, positive control treated with sesame oil (SO; vehicle), and injected *ia* with zymosan (100 μg/mice). Group 3, arthritic animals treated with 0.3 mg/kg/TMX; group 4, arthritic animals treated with 0.9 mg/kg/TMX; group 5, arthritic animals treated with 2.7 mg/kg//TMX; and group 6 received 17-β-estradiol (E2; 50 μg/kg) (2). All treatments with SO, TMX and E2 were performed for 5 consecutive days subcutaneously (*sc*).

Concomitantly, mice were submitted to ovariectomy and divided in 5 groups with 10 animals each as follows: group 1, positive control treated with SO; group 2, 3, and 4, arthritic animals treated with TMX at the doses described above, respectively, *sc* for 5 days; group 5, received hormone replacement with E2, for 5 consecutive days (*sc*).

For arthritis induction, animals received *ia* injection of zymosan at day 5, one hour after the last treatment with SO, TMX or E2.

### Ovariectomy

The mice were anesthetized intraperitoneally (*ip*) with a 10:1 mixture of ketamine (100 mg/kg) and xylazine (10 mg/kg) under aseptic conditions and were subjected to ovariectomy performed with a small incision along the dorsal midline. Both ovaries were excised, the dorsal wall was sutured and the cutaneous incisions closed with 10 mm clamps. Before and after surgery, mice received an injection (*ip*) of sodium diclofenac (5 mg/kg). The animals were allowed to recover for 2 weeks before treatments.

### Zymosan-induced arthritis

For experimental induction of arthritis by zymosan, mice were anesthetized by inhalation of halothane (0.01%) and were injected with zymosan (100 µg/mice, dissolved in 10 µL; Sigma, USA) in the right femoral tibial joint (3) and the contralateral joint was injected with an equal volume of saline (*ia*, negative control).

### Leukocyte migration

Four hours after arthritis induction, the animals were anesthetized (*ip*) with 100 mg/kg of ketamine and 10 mg/kg of xylazine (10:1) and euthanized by cervical dislocation. The knee joint was exposed and washed twice with 5-μL phosphate buffered saline (PBS) containing ethylenediaminetetraacetic acid (EDTA) diluted to a final volume of 100 μL with PBS/EDTA. The total number of leukocytes, diluted in the Turk solution, was determined in a Neubauer (New Optics, Germany) chamber under an optical microscope (Zeiss, Germany). Results are reported as the number of leukocytes per articular cavity. Differential cell counts were performed from articular exudate and smears obtained by cytocentrifuge (Cytospin 3-Shandon, Lipshaw Inc., USA). The cells were pelleted onto slides and then stained by hematoxylin-eosin dye for characterization of the leukocyte types according to their core-cytoplasm characteristics, using a 100× objective in immersion oil. In each slide, 100 cells were counted, differentiating three cell types: eosinophils, neutrophils and mononuclear cells. The quantification of each cell type was calculated from the percentage found in relation to the total number of cells.

### Analysis of the estrous cycle

The estrous cycle stage was assessed by examination of vaginal smears for two cycles of initiation and end of treatment. Briefly, exfoliated cell samples were obtained by lavage of the vagina with saline using a micropipette and then placing the sample on a glass slide. The cells were evaluated under an optical microscope to determine the phases of the estrous cycle (pro-estrus, estrus, diestrous and metaestrous) (11).

### Statistical analysis

Data are reported as means±SE. The results were statistically analyzed using one-way analysis of variance (ANOVA) followed by the Tukey's *post hoc* test. P<0.05 was considered statistically significant.

## Results

### Effect of TMX in neutrophil migration

**Figure 1.**
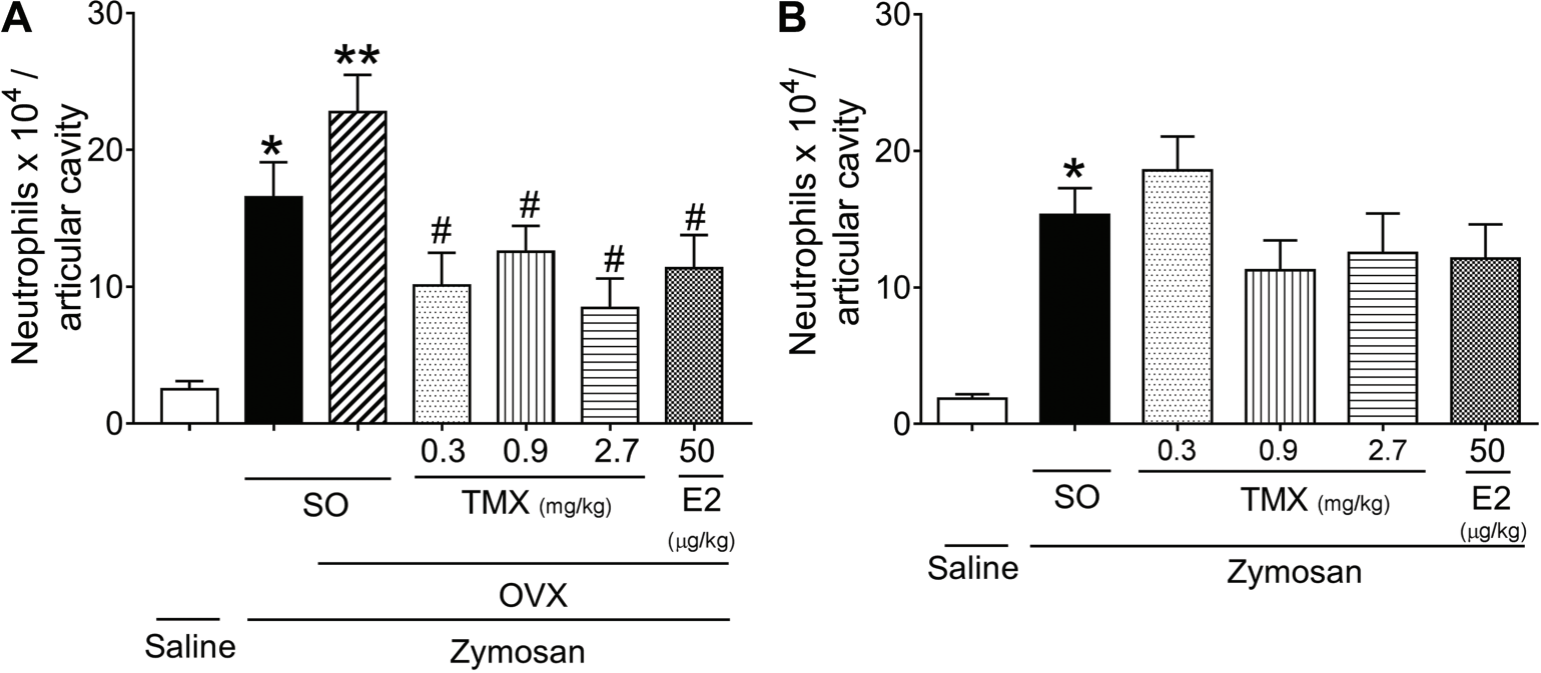
Effect of tamoxifen (TMX) and 17-β-estradiol (E2) on the neutrophil migration in zymosan-induced arthritis in mice. Ovariectomized (OVX, *Panel A*) or non-ovariectomized (*Panel B*) mice received treatment with sesame oil (SO; vehicle, *sc*), TMX (0.3, 0.9, and 2.7 mg/kg, *sc*) or E2 (50 μg/kg, *sc*) for 5 days. Another group received saline solution (*ia*). All groups were injected with zymosan (100 μg/mouse, *ia*), except the saline group. After 4 h, the leukocyte migration was evaluated in the articular cavity of the knee joint. Data are reported as means±SD. *P<0.05 *vs* saline group; **P<0.05 *vs* SO treatment and zymosan-injected mice; ^#^P<0.05 *vs* SO treatment and zymosan-injected OVX mice (ANOVA, Tukey's test).

Neutrophil migration into the articular cavity in OVX animals treated with TMX (0.3, 0.9, or 2.7 mg/kg) was significantly decreased compared with animals that received only SO. This effect was also demonstrated in the E2-treated group (Figure 1A).

In contrast, neutrophil migration in joint of animals without ovariectomy that were treated with TMX or E2 were not altered compared with mice that received SO (Figure 1B).

Therefore, TMX in OVX mice played agonist activity to effects of the endogenous hormone promoting neutrophil recruitment inhibition in the articular cavity of arthritic mice.

### Effect of tamoxifen on uterine weight

**Figure 2.**
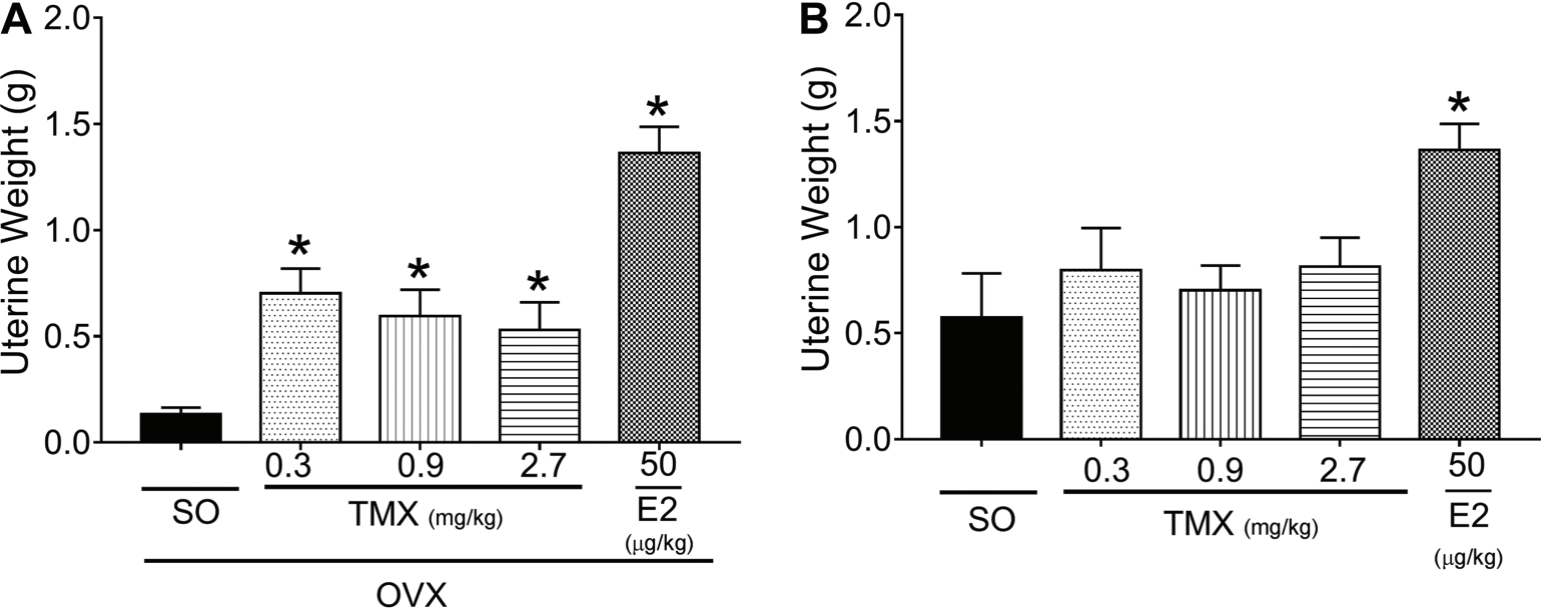
Effect of tamoxifen (TMX) and 17-β-estradiol (E2) on the uterine wet weight of mice. Ovariectomized (OVX, *Panel A*) or non-ovariectomized (*Panel B*) mice received treatment with TMX (0.3, 0.9 and 2.7 mg/kg, *sc*), E2 (50 μg/kg, *sc*) or sesame oil (SO; vehicle, *sc*) during 5 days. Data are reported as means±SD. *P<0.05 *vs* SO group (ANOVA, Tukey's test).

In OVX mice, treatment with TMX and E2 replacement promoted the increase of the uterus wet weight compared with SO-treated animals (Figure 2A).

However, in the presence of endogenous estrogen, TMX caused no change in the weight of the uterus of mice that received only SO compared with E2-treated animals (Figure 2B). These results confirmed the agonist TMX effect in relation to estrogen activity in OVX mice.

### Action of TMX in the estrous cycle of mice

As indicated in Table 1, there was no significant change in the estrous cycle of OVX mice without TMX treatment, predominating the metaestrous phase. However, five days after TMX treatment in OVX mice, there was a change in the predominant estrous cycle phase to estrus. Therefore, TMX in the absence of physiological estrogen promoted phase changes in the estrous cycle, corroborating our above-described results.

**Table 1.** Effect of TMX in the estrous cycle of ovariectomized mice.

Experimental groups	Evaluation of the estrous cycle before treatments			Evaluation of the estrous cycle after treatments		
SO	Met.	Met.	Met.	Met.	Met.	Diest.
E2 (50 µg/kg)	Diest.	Met.	Met.	Est.	Est.	Est.
TMX						
0.3 mg/kg	Met.	Met.	Met.	Pro.	Pro.	Est.
0.9 mg/kg	Diest.	Met.	Met.	Est.	Est.	Est.
2.7 mg/kg	Met.	Met.	Met.	Est.	Est.	Est.

The experimental groups consisted in ovariectomized mice treated with 17β-estradiol (E2, 50 µg/kg, *sc*), different doses of tamoxifen (TMX, 0.3, 0.9, and 2.7 mg/kg, *sc*) or only with sesame oil (SO, vehicle, *sc*). The estrous cycle stage was evaluated before and after treatments and determined in pro-estro (Pro.), estrus (Est.), diestrous (Diest.), and metaestrous (Met.).

## Discussion

Some diseases accelerate in postmenopausal women. This relationship is very pronounced in arthritis, in which estrogen depletion increases the propensity for arthritis (2).

Hormone replacement therapy (HRT) is an intervention to relieve some symptoms of decreased hormone levels. A clinical study evaluating the effect of HRT with estrogen on arthritis indicated an anti-inflammatory effect, reducing joint damage and increasing bone density. However, the prolonged use of HRT increases the incidence of thromboembolism, heart attack and breast cancer. It has been reported that compounds with physiological activity and estrogen-like chemical structure can attenuate climacteric problems and guarantee a better quality of life for patients (7,10).

SERMs are drugs with a chemical structure similar to estrogen that can interact with the estrogen receptor and modulate agonist or antagonist responses (6). TMX, one of the first SERMs available, caused estrogen antagonistic action in inflammatory diseases such as systemic lupus erythematosus in the experimental model of female NZB/NZW mice, by inhibiting the differentiation of Th1 and Th17 cells and suppression of pro-inflammatory cytokines, such as IL-17 and TNF-α (12). It is known that in arthritis, IL-17-producing CD4 T-helper cells (Th17 cells) are present in the inflamed joint cavity and contribute to the progression of an early inflammation to persistent chronic arthritis (13).

Interestingly, in the present study, TMX treatment was found to significantly reduce zymosan-induced arthritis in OVX mice, promoting the inhibition of neutrophil recruitment in the absence of estrogen. In addition, it has been confirmed that treatment with 17β-estradiol suppressed the progression of inflammation in arthritis (14). TMX can modulate NF-κβ factor by regulating immune function through the transcription of inflammatory mediators, closely related to the estrogen receptor (15). Thus, these data strongly demonstrated the agonist properties of this SERM in the estrogen receptor.

To understand the action of TMX in OVX mice some points must be mentioned. First, the most important features of all SERMs are that they have less agonistic effects on the reproductive system in the presence of E2 (16). Second, the agonist action of TMX occurs because of a large, flexible binding bag containing key amino acids (Glu353, Arg394 and His524) that allows binding of several ligands in the estrogen receptor, where the E2 interaction is rigid and has high affinity. In contrast to E2, TMX has a bulky side chain in its structure that prevents connection in the connection pocket in the presence of E2, hindering the interaction dynamics (17,18).

The results of uterine weight analysis corroborate previous evidence, in which the absence of estrogen caused by ovariectomy promotes reduction of uterine weight (19). In contrast, when TMX was administered to OVX mice, it attenuated the effect of castration and promoted a discrete but significant increase in uterine weight similar to estrogen administration, confirming the agonist effect of TMX in the uterus. These findings were consistent with reports of elevated uterine weight promoted by TMX in OVX collagen-induced arthritis (20).

In addition, OVX animals treated with different doses of TMX were stable at the estrus phase of the estrous cycle. This is consistent with previous observations, which demonstrated that the E2 level is increased in this phase, similar to physiological conditions (11).

In conclusion, this study demonstrated the TMX agonist effect in inhibiting the migration of inflammatory cells and preventing the arthritis progression in OVX mice. Therefore, these findings should be considered in future research with models of chronic arthritis, with the aim of evaluating TMX in the treatment of postmenopausal diseases.

## References

[1] Ishizuka M, Hatori M, Suzuki T, Miki Y, Darnel AD, Tazawa C (2003). Sex steroid receptors in rheumatoid arthritis. Clin Sci.

[2] Hong-Fang L, Duan Y, Wang L, Tian ZF, Qiu XQ, Zhang Y (2013). Effects of estrogen and phytoestrogens on endometrial leakage in ovariectomized rats and the related mechanisms. Acta Physiol Sin.

[3] Yamada NA, Grespan R, Yamada AT, Silva EL, Silva-Filho ES, Damião MJ (2013). Anti-inflammatory activity of *Ocimum americanum* L. essential oil in experimental model of zymosan-induced arthritis. Am J Chinese Med.

[4] Komi J, Möttönen M, Luukkainen R, Lassila O (2001). Non-steroidal anti-oestrogens inhibit the differentiation of synovial macrophages into dendritic cells. Rheumatology.

[5] Andersson A, Bernardi AI, Stubelius A, Nurkkala-Karlsson M, Ohlsson C (2016). Selective oestrogen receptor modulators lasofoxifene and bazedoxifene inhibit joint inflammation and osteoporosis in ovariectomised mice with collagen-induced arthritis. Rheumatology.

[6] Liu JH (2017). Is there a SERM in your menopause tool kit?. Menopause.

[7] Moon Z, Hunter MS, Moss-Morris R, Hughes LD (2016). Factors related to the experience of menopausal symptoms in women prescribed tamoxifen. J Psychosom Obstet Gynaecol.

[8] Turner RT, Wakeley GK, Hannon KS, Norman HB (1987). Tamoxifen prevents the skeletal effects of ovarian hormone deficiency in rats. J Bone Miner Res.

[9] Morita M, Sato Y, Iwasaki R, Kobayashi T, Watanabe R, Oike T (2016). Selective estrogen receptor modulators suppress Hif1alpha protein accumulation in mouse osteoclasts. PloS One.

[10] MacDonald AG, Murphy EA, Capell HA, Bankowska UZ, Ralston SH (1994). Effects of hormone replacement therapy in rheumatoid arthritis: a double-blind placebo-controlled study. Ann Rheum Dis.

[11] Gal A, Lin PC, Barger AM, Mac Neill AL, Ko C (2014). Vaginal fold histology reduces the variability introduced by vaginal exfoliative cytology in the classification of mouse estrous cycle stages. Toxicol Pathol.

[12] Sthoeger ZM, Zinger H, Mozes E (2003). Beneficial effects of the anti oestrogen tamoxifen on systemic lupus erythematosus of (NZBxNZW) F1 female mice are associated with specific reduction of IgG3 autoantibodies. Ann Rheum Dis.

[13] Paulissen SMJ, van Hamburg JP, Dankers W, Lubberts E (2015). The role and modulation of CCR6+ Th17 cell populations in rheumatoid arthritis. Cytokine.

[14] Holmdahl R, Jansson L, Meyerson B, Klareskog L (1987). Oestrogen induced suppression of collagen arthritis: I. Long term oestradiol treatment of DBA/1 mice reduces severity and incidence of arthritis and decreases the anti type II collagen immune response. Clin Exp Immuno.

[15] Sweeney SE, Firestein GS (2004). Rheumatoid arthritis: regulation of synovial inflammation. Int J Biochem Cell Biol.

[16] Borjesson AE, Farman HH, Moverare-Skrtic S, Engdah C, Antal MC, Koskela A (2016). SERMs have substance specific effects on bone and these effects are mediated via ERaAF-1 in female mice. Am J Physiol Endocrinol Metab.

[17] Gronemeyer H, Gustafsson JA, Laudet V (2004). Principles for modulation of the nuclear receptor superfamily. Nat Rev Drug Discov.

[18] Martinkovich S, Shah D, Planey SL, Arnott JA (2014). Selective estrogen receptor modulators: tissue specificity and clinical utility. Clin Interv Aging.

[19] Black LJ, Sato M, Rowley ER, Magee DE, Bekele A, Williams DC (1994). Raloxifene (LY139481 HCI) prevents bone loss and reduces serum cholesterol without causing uterine hypertrophy in ovariectomized rats. J Clin Invest.

[20] Wood CE, Kaplan JR, Fontenot MB, Williams JK, Cline JM (2010). Endometrial profile of tamoxifen and low-dose estradiol combination therapy. Clin Cancer Res.

